# Pre-clinical safety and efficacy evaluation of *Helicobacter* *Pylori* neutrophil-activating protein (NAP)-armed CAR-T cells targeting B cell lymphomas

**DOI:** 10.1007/s00262-025-04112-1

**Published:** 2025-07-12

**Authors:** Jing Ma, Tina Sarén, Chuan Jin, Hyeong Su Kim, Paola Donaji Contreras Pineda, Marina de Bernard, Rose-Marie Amini, Veronica Rondahl, Gunilla Enblad, Di Yu, Magnus Essand

**Affiliations:** 1 Department of Immunology, Genetics and Pathology, Uppsala University, Uppsala, Sweden; 2 Department of Biology, University of Padova, Padova, Italy; 3 Department of Animal Biosciences, Division Anatomy, Physiology, Immunology and Pathology, Swedish University of Agricultural Sciences, Uppsala, Sweden; 4 Science for Life Laboratory, Uppsala University, Uppsala, Sweden; 5 Present Address: Hallym University Medical Center, Seoul, South Korea

**Keywords:** CD20, Chimeric antigen receptor, CAR-T cell, Neutrophil-activating protein, Safety, Toxicity, Lymphoma, Cytokine release, Rituximab

## Abstract

CD19 CAR-T cell therapy shows striking results in treating B cell malignancies. However, approximately two-thirds of the lymphoma patients eventually relapse, with about one-third displaying CD19-negative tumors at relapse. Our previous study showed that CAR-T cells armed with the *Helicobacter* *pylori* neutrophil-activating protein (NAP), CAR(NAP)-T cells, can trigger a bystander immune response and eliminate CAR-target-antigen-negative tumor cells. Here, we report the development of CD20-targeted CAR-T cells (CAR20-T cells), with the targeting moiety from rituximab, and the safety and efficacy of NAP-armed CAR-T cells. CAR20-T cells displayed efficient and specific cytotoxic potential against multiple human B cell lymphoma cell lines in vitro. In addition, primary mantle cell lymphoma cells, isolated from a patient who relapsed after rituximab treatment, can also be eliminated by CAR20-T cells. CAR20(NAP)-T cells delayed tumor growth and prolonged survival of mice with lymphoma. No obvious histopathological alteration in major organs were observed in mice treated with CAR(NAP)-T cells. Further, no excessive cytokine release or immune cell activation was observed when human blood from healthy volunteers was exposed to recombinant NAP protein in an ex vivo blood loop assay, suggesting a safe therapeutic profile for NAP. Taken together, these results warrant the clinical investigation of CAR20(NAP)-T cells.

**Supplementary Information:**

The online version contains supplementary material available at 10.1007/s00262-025-04112-1.

## Introduction

Cancer immunotherapy has demonstrated remarkable advances in the last decade and chimeric antigen receptor (CAR)-T cell therapy has played a key role in this success. CD19-targeting CAR-T cell therapy has been successfully implemented against therapy-resistant B cell malignancies with several products being approved in the US, EU  and China. The results have been especially striking when targeting acute lymphoblastic leukemia (ALL) [1, 2], and lymphomas [3–6]. Despite the encouraging outcomes, approximately two-thirds of large B cell lymphoma patients will eventually relapse after CD19 CAR-T cell therapy, with about one-third experiencing CD19-negative tumors upon relapse [7–11].

CD20 is expressed on B cells throughout most of their development and expression is retained in B cell lymphomas. CAR-T cells against CD20, with the targeting moiety from the antibody clone Leu16, were among the first being developed and evaluated clinically [12, 13]. The CD20-targeting antibody rituximab was the first monoclonal antibody to be approved in oncology with excellent results for patients suffering from B cell malignancies [14, 15]. Rituximab together with cyclophosphamide, hydroxydaunorubicin, oncovin and prednisone, the so-called R-CHOP regimen, is the first-line treatment for several types of B cell lymphomas [16–19]. At relapse, loss of CD20 or mutation of the rituximab targeting epitope is rarely the reason for failure of R-CHOP treatment [20]. Therefore, the rituximab targeting epitope of CD20 could still be targeted using a different therapeutic approach including CAR-T cells. In fact, rituximab-derived CD20-targeting CAR-T cell constructs have been developed and tested pre-clinically [21]. Through rational protein design of the signaling domain, a construct was successfully identified with superior anti-tumor effect in comparison to the FMC63 clone-derived CD19 targeting CAR-T cells [21].

The *Helicobacter* *pylori* neutrophil-activating protein (NAP) is a major virulence factor of *Helicobacter* *pylori*. NAP promotes a T helper (Th) 1 polarized immune response, which is favorable in cancer treatment [22–24]. Also, NAP acts as a chemoattractant for innate immune cells, such as neutrophils and monocytes [25] and can both recruit and activate dendritic cells (DCs) [26]. We have shown that NAP, when released from NAP-armed CAR(NAP)-T cells, can be used as an immunomodulator to promote epitope spread and induce endogenous T cell responses towards CAR-target-antigen-negative tumor cells [27].

The current study aimed to evaluate the toxicity of NAP-armed CAR-T cells and their feasibility of targeting human CD20. CAR(NAP)-T cells targeting CD20 have the potential to treat B cell lymphomas irrespective of prior CD19 CAR-T cell therapy and may prevent subsequent CAR-antigen-negative relapses. We show that CAR20-T cells displayed efficient and specific cytotoxicity in vitro against human lymphoma cell lines and patient-derived mantle cell lymphoma cells. Further, CAR20-T cells engineered to secrete NAP (CAR20(NAP)-T cells) exhibit enhanced anti-tumor efficacy and can significantly prolong survival of mice with lymphoma. CAR20(NAP)-T cells have the capability to target circulating healthy B cells and thereby release NAP systemically. Given that the rituximab-derived CAR20(NAP)-T cells specifically target human CD20, we evaluated the potential systemic toxicity of NAP using NAP-armed CAR-T cells directed against murine CD19 in vivo. Our findings indicate that these CAR(NAP)-T cells are safe and do not induce excessive cytokine release. Additionally, recombinant NAP protein induced lower cytokine release and lymphocyte activation in human blood when compared to Alemtuzumab, which is used safely in the clinic. In conclusion, it is feasible to target human CD20, and arming CAR-T cells with NAP was shown to be safe.

## Results

### CAR20-T cells efficiently kill CD20-expressing lymphoma cells in vitro

**Fig. 1:**
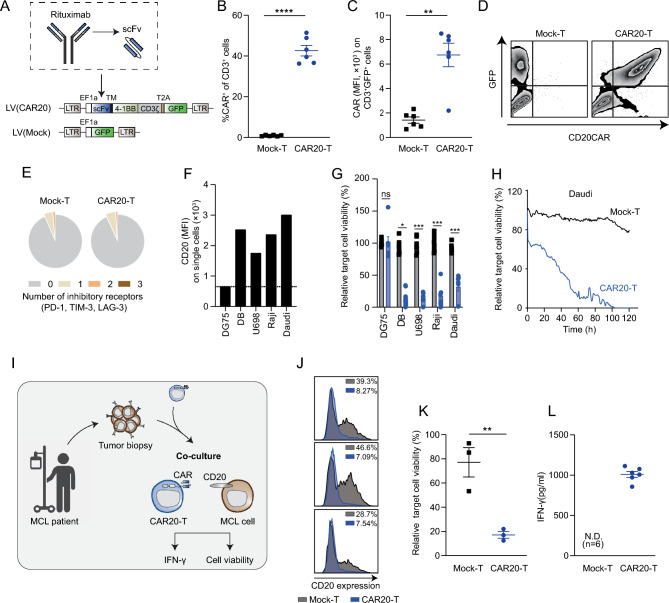
Human CAR20-T cells displayed potent cytotoxic capacity towards CD20-expressing lymphoma cells in vitro. **A** Lentiviral (LV) constructs used for human T cell engineering. LV(CAR20) containing the single chain variable fragment (scFv) derived from rituximab and 4-1BB and CD3ζ intracellular domains together with green fluorescent protein (GFP) for detection of transduced T cells. LV(Mock) only encoding GFP was used as control. **B** Transduction efficiency of CAR20-T presented as percentage of CAR^+^ cells out of CD3^+^ cells. Mock-T cells were used as control. **C** Mean fluorescent intensity (MFI) of CAR on CAR20-T (CD3^+^GFP^+^) and Mock-T (CD3^+^GFP^+^) cells. Each dot represents T cells isolated from one healthy donor (n = 6), with bars indicating mean ± SEM. Non-parametric unpaired t-test was used to compare between groups (**: p < 0.01, ****: p < 0.0001). **D** Representative contour plots of the concordance between GFP and CAR expression in the CD3^+^ population of CAR20-T and Mock-T cells. **E** Proportion of CAR20-Ts or Mock-Ts (CD3^+^GFP^+^) expressing either 0, 1, 2 or 3 activation/inhibitory receptors (PD-1, TIM-3 and LAG-3) (n = 3). **F** CD20 expression on five human B cell lymphoma cell lines, DG75, DB, U698, Raji and Daudi. **G** Relative viability of luciferase-tagged lymphoma target cells after 4 days exposure to CAR20-T cells at the effector to target ratio = 3:1. Each dot represents T cells isolated from one healthy donor (n = 8), with bars indicating mean ± SEM. Non-parametric unpaired t-test was used to compare groups for each cell line (*: p < 0.05, ***: p < 0.001). **H** Relative viability of scarlet-expressing Daudi cells measured every hour over time by total integrated intensity using Incucyte after exposure to CAR20-T cells or Mock-T cells, prepared from one healthy donor, at the effector to target ratio 5:1. **I–L** Primary mantle cell lymphoma (MCL) cells, isolated from a patient who had developed resistance towards rituximab (anti-CD20 antibody) treatment, were co-cultured with either CAR20-T cells or Mock-T cells at a 1:1 effector to target cell ratio for 3 days. **I** Illustration of experimental set up. **J** Histogram displaying CD20^+^ tumor cells after co-culture with CAR20-T or Mock-T cells. Each individual histogram represents a co-culture of lymphoma cells and transduced T cells derived from one donor. **K** Quantification of the percentage of CD20^+^ tumor cells in panel **J**. Each dot represents T cells isolated from one healthy donor (n = 3) bars indicating mean ± SEM. Non-parametric unpaired t-test was used to compare groups (**: p < 0.01). **L** IFN-γ production from T cells as in panel **J**. (N.D. = not detectable)

Human CAR20-T cells containing a single chain variable fragment (scFv) derived from rituximab, and the intracellular activation domains from 4-1BB (CD137) and CD3ζ were generated using a lentiviral vector (Fig. 1A). The construct also contained green fluorescent protein (GFP) allowing detection of transduced T cells (Fig. 1A). T cells transduced with a lentiviral vector containing only GFP (termed Mock-T cells) were used as negative controls (Fig. 1A). Upon transduction, CAR20 was expressed on the T cell surface (Fig. 1B, C) and correlated with GFP expression (Fig. 1D). It has been shown that CARs, due to their intrinsic design, may induce antigen-independent tonic signaling and subsequent CAR-T cell exhaustion [28, 29]. Therefore, we evaluated if CAR20-T cells displayed phenotypic exhaustion during in vitro culture. CAR20-T cells, as Mock-T cells, were mainly negative for inhibitory markers (Fig. 1E), indicating that the design of the CD20-targeting CAR does not cause excessive antigen-independent tonic signaling and CAR-T cell exhaustion.

To investigate if CAR20-T cells could specifically kill malignant B cell lines in vitro, CAR20-T cells were co-cultured with CD20^+^ (DB, U698, Raji, Daudi) and CD20^−^ (DG75) lymhoma cell lines (Fig. 1F). CAR20-T cell killing was specific as all CD20-expressing lymphoma cells were killed, while the CD20^−^ lymphoma cell line DG75 was not (Fig. 1G, H**; **Supplementary Figure S1A, B). To assess clinical relevance, CAR20-T cells were co-cultured with primary mantle cell lymphoma (MCL) cells isolated from a tumor biopsy of a patient that had become refractory to rituximab treatment (Fig. 1I). Encouragingly, CAR20-T cells were able to efficiently kill CD20-expressing MCL cells and release high amounts of IFN-γ, as compared to Mock-T cells (Fig. 1J-L).

In conclusion, CAR20-T cells could successfully be produced without induction of antigen-independent exhaustion in vitro. CAR20-T cells displayed potent cytotoxicity towards all tested CD20^+^ malignant B cell lines as well as primary CD20^+^ MCL cells in vitro.

### NAP-armed CAR-T cells targeting CD20 exhibited enhanced anti-tumor efficacy

**Fig. 2:**
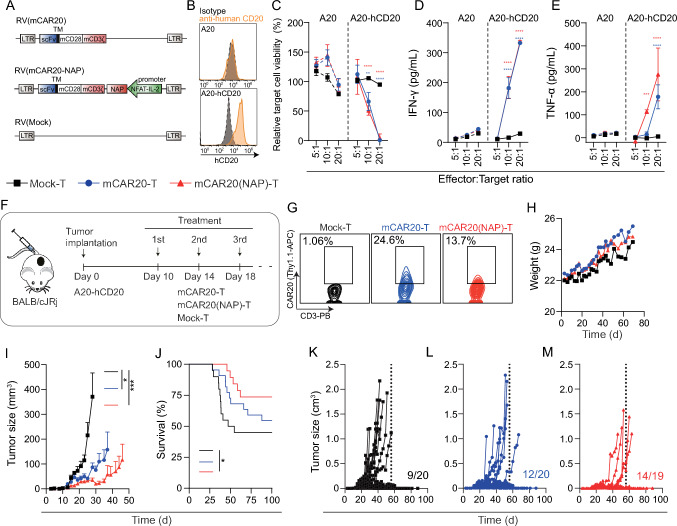
Murine mCAR20-T and mCAR20(NAP)-T cells display cytotoxicity against murine lymphoma engineered with human CD20, A20-hCD20, in vitro and display therapeutic efficacy in vivo. **A** Schematic illustration of retroviral (RV) constructs used for murine T cell engineering. **B** Histogram displaying the expression of human (h)CD20 on parental A20 and cells engineered to express human CD20 (A20-hCD20). **C** Relative target cell viability, **D** IFN-γ, and **E** TNF-α secretion in the co-culture where engineered mouse T cells were exposed to luciferase-tagged parental A20 or A20-hCD20 cells at effector to target cell ratios 5:1–20:1 for 4 days. The experiment was performed twice. Data is shown as mean ± SD from one representative co-culture experiment with experimental triplicates. Two-way ANOVA with Tukey’s multiple comparisons test was used to compare between groups. Statistical significance is color-coded between corresponding treatment group and Mock group (**: p < 0.01; ****: p < 0.0001). **F–M** Mice were implanted subcutaneously with 2 × 10^5^ A20-hCD20 cells and treated three times (3 × 10^6^ T cells/dose) with Mock-T (n = 20), mCAR20-T (n = 20) or mCAR20(NAP)-T (n = 19). **F** Illustration of treatment schedule. **G** representative contour plot showing CAR expression level (represented as Thy1.1) in CD3^ +^ cells. All experiments were assessed using a mixed population containing both transduced and untransduced cells. **H** Weight (mean) of mice after tumor implantation and during treatment. **I** Tumor size (mean + SEM). **J** Mouse survival (Kaplan–Meier) curve, and **K–M** tumor size for individual mice after treatment are presented. Numbers in **K–M** indicate tumor-free mice. The experiment was performed twice, and data was pooled. Tumor sizes on day 28 (time when the first mouse was sacrificed due to reaching the humane endpoint in Mock-T group) were compared between each treatment group using nonparametric Kruskal–Wallis One-way ANOVA test with Dunn’s correction for multiple comparisons. The survival curves were compared by the Log-rank test (*: p < 0.05, ***: p < 0.001)

To evaluate the therapeutic efficacy of CAR20-T cells in an immunocompetent mouse model, murine (m)CAR-T cells were engineered using retroviral vectors (RV) with the CAR20 linked to the murine CD28 and CD3ζ intracellular signaling domains (Fig. 2A**).** We have previously shown that NAP-armed CAR-T cells improve therapeutic efficacy in several murine tumor models compared to conventional CAR-T cells [27]. To evaluate if NAP-armed mCAR20-T cells could improve therapeutic efficacy, a retrovirus vector for NAP-armed mCAR20-T cells was also constructed (Fig. 2A). As the rituximab-derived CAR20 only binds to human CD20, the murine B cell lymphoma A20 cell line was engineered to express human CD20 (Fig. 2B), termed A20-hCD20, to allow evaluation of mCAR20-T cell treatment. First, the cytotoxic capacity of mCAR20-T cells was evaluated in vitro by co-culturing mCAR20-T, mCAR20(NAP)-T and Mock-T cells with A20-hCD20 tumor cells at different effectors to target cell ratios. Both mCAR20-T and mCAR20(NAP)-T cells could efficiently kill the A20-hCD20 target cells (Fig. 2C) and secreted IFN-γ (Fig. 2D) and TNF-α (Fig. 2E) upon antigen encounter. Neither killing nor IFN-γ or TNF-α secretion were detected against antigen-negative A20 cells (Fig. 2C-E). There was no apparent difference between mCAR20-T and mCAR20(NAP)-T cells in terms of cytotoxicity and cytokine secretion. These data indicated that insertion of NAP did not negatively affect the in vitro antitumor efficacy, which is in accordance with our previous findings [27]. Further, in a xenograft setting, systemically injected human CAR20(NAP)-T cells slowed down tumor growth, prolonged survival and cured some mice, supporting in vivo efficacy of CAR20(NAP)-T cells (Supplementary Figure S2A-C).

To fully evaluate the potency of NAP-armed CAR20-T cells in vivo, we investigated the therapeutic efficacy in an immunocompetent mouse model with subcutaneous A20-hCD20 lymphoma. Mice were treated (Fig. 2F) intravenously (i.v.) with mCAR20-T, mCAR20(NAP)-T or Mock-T cells (Fig. 2G) 10-, 14-, and 18-days post tumor implantation. No signs of toxicity or adverse health effects were observed after treatment and the mice maintained a stable weight during treatment (Fig. 2H). Nearly half of the mice became tumor-free in the Mock-T treated group (Fig. 2I-M), possibly due to immunogenicity of human CD20. Nevertheless, mCAR20-T or mCAR20(NAP)-T treatment improved the therapeutic outcome by significantly delaying tumor growth compared to Mock-T treatment (Fig. 2I). Further, mCAR20(NAP)-T significantly prolonged the survival compared to Mock-T treatment (Fig. 2J). In addition, there were more tumor free mice after both mCAR20-T and mCAR20(NAP)-T treatment in comparison to Mock-T cell treatment (Fig. 2K–M).

### Treatment-related toxicity was not observed in CAR(NAP)-T cell treated mice harboring lymphoma

**Fig. 3:**
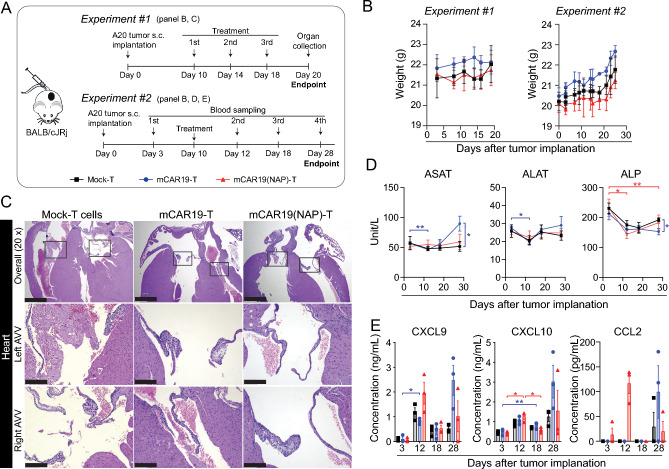
Inclusion of NAP in CAR-T cells is unlikely to compromise safety in vivo. **A** Illustration of experimental set up. **B** Weight of mice after tumor implantation and treatment in both experiments. Data is shown as mean ± SD (n = 3). **C** Representative images of Hematoxylin–Eosin (HE) stained heart tissue sections collected from each treatment group, showing mild myxomatous degeneration of cardiac valves. Other examined organs had no lesions (Supplementary Fig. 3A-E). AVV: Atrioventricular valves. Scale bar equals 1 mm in 20 × images and equals 200 µm in zoomed-in images. **D** Liver enzymes (ASAT, ALAT, ALP) measured at different time points after treatment. Data is shown as mean ± SD (n = 3). ASAT: aspartate aminotransferase; ALAT: alanine aminotransferase; ALP: alkaline phosphatase. Two-way ANOVA with Geisser-Greenhouse correction and Dunnett’s multiple comparison tests were performed to compare matched values between time points (within the same treatment group) and values between groups (at same time point). The statistical labels are colored according to treatment groups (*: p < 0.05; **: p < 0.01). **E** Systemic cytokine levels measured at different time points after treatment. A panel of 13 cytokines (LEGENDplex) was assessed and the cytokines with detectable level are presented (CXCL9, CXCL10, CCL2). Each dot represents an individual animal with bars indicating mean ± SEM (n = 3). Two-way ANOVA with Geisser-Greenhouse correction and Dunnett’s multiple comparison tests were performed to compare matched values between time points (within the same treatment group) and values between groups (at the same time point). The statistical labels are colored according to treatment groups (*: p < 0.05; **: p  < 0.01)

There were no detectable side effects in mice with A20-hCD20 tumors upon treatment with CAR20(NAP)-T, and a stable body weight was maintained (Fig. 2H). However, as rituximab-derived CAR20(NAP)-T cells only bind human CD20, NAP will only be released locally at the A20-hCD20 tumor site. In patients, CAR20(NAP)-T cells would release NAP into the circulation upon recognition of healthy B cells and therefore the safety of broader release of NAP must be evaluated. We therefore treated A20 (CD19^+^) lymphoma-bearing mice with mCAR19-T and mCAR19(NAP)-T cells, targeting murine CD19 in both A20 and normal mouse B cells (Fig. 3A). After treatment, major organs including heart, kidney, liver, lung, brain, and spleen were collected for histopathological evaluation to identify potential tissue damage. Systemic cytokine release and liver function were also assessed for potential toxicities (Fig. 3A). No signs of toxicity or ill health were observed in mice treated with either mCAR19-T or mCAR19(NAP)-T cells and the body weight of these mice remained stable throughout the study (Fig. 3B). There were no macroscopic or histopathologic lesions in the kidney, liver, lung, brain, or spleen after treatment (Supplementary Figure S3A-E). In all treatment groups, including the Mock-T cell treated animals, there were mild lesions in the heart consistent with early myxomatous degeneration of cardiac valves (Fig. 3C). Therefore, it is unlikely that the lesions were associated with systemic release of NAP. Liver enzymes including aspartate aminotransferase (ASAT), alanine aminotransferase (ALAT), and alkaline phosphatase (ALP) were also evaluated (Fig. 3D). Even though some significant differences were observed between groups/timepoints, the levels of these enzymes were not fluctuating more than 2 folds (Fig. 3D). The ASAT level remained stable throughout the experiment with essentially no differences between treatment groups except a slight increase in mCAR19-T cell treated mice on day 28 compared to Mock-T treated mice (Fig. 3D). No differences in ALAT levels were observed between the groups at any given time point (Fig. 3D). The ALP level in all treated groups showed a slight decrease initially, though no big difference was observed between different treatment groups (Fig. 3D). When analyzing a panel of 13 cytokines (IFN-γ; IL-10; CCL4; IFN-α; CXCL9; CXCL10; TNF-α; IL-6; VEGF; IL-4; CCL3; CCL2; GM-CSF) in blood, only CXCL9, CXCL10 and CCL2 were detectable (Fig. 3E). No clear pattern of systemic cytokines was noticed, while there might be a trend indicating that mCAR19(NAP)-T induce cytokine (CXCL9 and CCL2) release earlier than mCAR19-T not armed with NAP. Overall, no systemic cytokine release related toxicity was observed in the tested model. In conclusion, arming the CAR-T cells with NAP did not change the safety profile of CAR-T cell treatment.

### Recombinant NAP protein does not cause toxicity in human blood

**Fig. 4:**
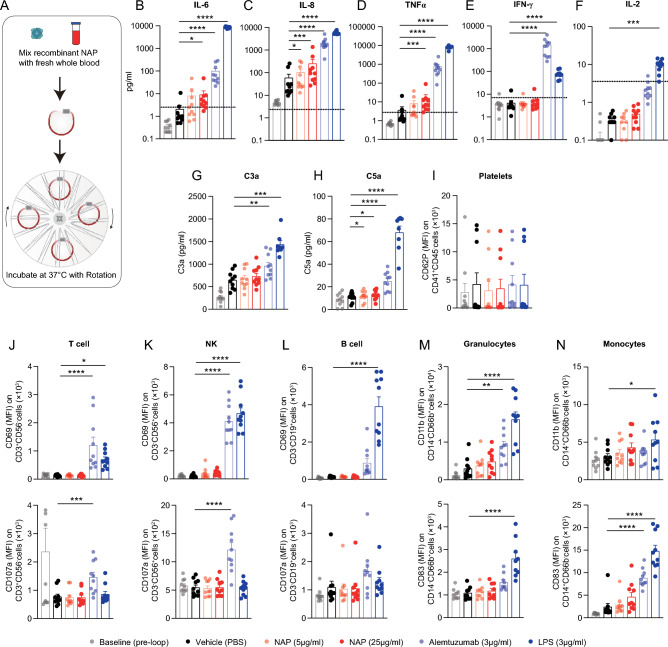
Recombinant NAP protein did not induce blood immune cell activation or cytokine release at levels above unmanageable clinical toxicity in the rotating whole human blood loop system. Recombinant NAP protein was evaluated for potential excessive immune activation side-by-side with Alemtuzumab, which has a manageable toxicity profile in the clinic. **A** Freshly acquired human blood was incubated in the rotating heparin-coated loops with either Phosphate-buffered saline (PBS, negative control), NAP, Alemtuzumab or Lipopolysaccharide (LPS, positive control) for immune activation. **B–F** Cytokines levels after 4 h incubation (n = 10), **B** IL-6, **C** IL-8, **D** TNFα, **E** IFN-γ and **F** IL-2 were analyzed by MSD Discovery® multiplex. The calculated lower limit of quantification (LLOQ) was marked with a dotted line. Each donor was run twice on separate occasions. **G** C3a and **H** C5a levels in 15 min plasma samples measured by ELISA. **B–H** paired Student’s t-test was performed on Log_10_ transformed values (values below LLOQ were set up to LLOQ) with Holm-Sidak correction for multiple comparisons between PBS and other groups. **I** Mean fluorescent intensity (MFI) of CD62P on platelets (CD41^+^CD45^−^). **J–L** MFI of CD69 and CD107a on **J** T cells (CD3^+^CD56^−^), **K** NK cells (CD3^−^CD56^+^) and **L** B cells (CD3^−^CD19^+^). **M–N** MFI of CD11b and CD83 on **M** granulocytes (CD14^−^CD66b^+^) and **N** monocytes (CD14^+^CD66B.^−^). **J–N** Each dot represents data from a healthy donor (n = 10), with bars indicating mean + SEM. One-way ANOVA was performed with Dunnett’s correction for multiple comparisons between PBS and other groups (* = p < 0.05, **: p < 0.01, ***: p < 0.001, ****: p < 0.0001)

In a clinical setting, NAP would be secreted systemically if CAR20(NAP)-T cells encountered healthy B cells in the blood of treated patients. We therefore sought to investigate the potential toxicity caused by systemic release of NAP. Recombinant NAP protein was incubated into a whole human blood loop system [30] to investigate if and to which extent NAP induces cytokine release, complement activation, and immune cell activation in fresh circulating blood from healthy human donors (Fig. 4A). Two concentrations of NAP protein, 5 and 25 µg/ml, were evaluated in the blood loop system. Alemtuzumab (anti-CD52, 3 µg/ml) was used as reference control since it is used for treatment of B cell chronic lymphocytic leukemia with manageable adverse events, also with respect to cytokine release [31, 32]. Lipopolysaccharide (LPS) was used as a positive activating control as it is one of the most potent innate immune activating stimuli that triggers strong release of TNFα and IL-6 from innate cells such as monocytes [33].

We observed an increased level of IL-6 over PBS control with an average of 3.9-fold increase at 5 μg/ml of NAP and 4.3-fold increase (*p* < 0.05) at 25 μg/ml of NAP (Fig. 4B). The levels of IL-8 and TNFα were increased compared to PBS control at both NAP concentrations, with an average of 1.7-fold and 4.3-fold increase of IL-8 (*p* < 0.05 at 5 μg/ml of NAP and p < 0.001 at 25 μg/ml of NAP) and an average of 2.3-fold and 4.4-fold increase of TNFα (p < 0.001 at 25 μg/ml of NAP) (Fig. 4C, D). Neither concentration of NAP induced quantifiable level of IFN-γ and IL-2 in human blood, as all values were below the lower limit of quantification (LLOQ) (Fig. 4E, F). In all cases, NAP induced less cytokine release than the reference Alemtuzumab (Fig. 4B–F). Additionally, we observed a modest increase of complement activation with cleaved C3a for NAP compared to PBS control (Fig. 4G), while cleaved C5a was significantly increased at both NAP concentrations (p < 0.05) (Fig. 4H). On the other hand, the level of complement activation was far less compared to Alemtuzumab (Fig. 4G, H). This indicates that NAP, at the tested concentration, did not activate the complement system to a functional level as compared to Alemtuzumab.

Next, we analyzed the ability of recombinant NAP protein to induce the activation of human immune cells. Platelet activation was assessed based on the level of CD62P (P-selectin) on CD41^+^CD45^−^ cells (Fig. 4I). The expression levels of CD69 and CD107a were used to evaluate the activation of T cells (CD3^+^CD56^−^), NK cells (CD3^−^CD56^+^) and B cells (CD3^−^CD19^+^) (Fig. 4J–L). The expression levels of CD83 and CD11b were used to assess the activation of monocytes (CD14^+^CD66b^−^) and granulocytes (CD14^−^CD66b^+^) (Fig. 4M, N). In short, we did not see any significant NAP-mediated activation of platelets, T cells, B cells, NK cells, monocytes or granulocytes at either of the two analyzed concentration levels (Fig. 4I–N). Alemtuzumab activated T cells, NK cells and granulocytes (Fig. 4J, K, M). None of the treatments affected blood cell counts (Supplementary Figure S4A-H). In conclusion, NAP induced concentration-dependent cytokine release and a slight complement activation. The activation levels were lower than the reference Alemtuzumab, whose side-effects are manageable in the clinic.

## Discussion

CD19 CAR-T cell therapy has shown successful results against relapsed/refractory (r/r) B cell malignancies [3, 7, 34]. However, follow-up has shown that about two-thirds of lymphoma patients acquire secondary resistance which is often associated with downregulation or loss of CD19 [7, 10]. CD20 has been proposed as an alternative CAR-target for treatment of large B cell lymphoma (LBCL) as it remains expressed after CD19 CAR-T cell therapy [10]. In addition, CD20 alterations or losses are rare in LBCL despite the usage of anti-CD20 antibody therapy in standard of care [10, 20].

Several different CD20-targeting CAR-T cells have been developed by pioneers in the field, wherein different targeting moieties were used, including the HB-9645 hybridoma derivative scFv [35, 36], the Leu16 anti-CD20 antibody clone derived scFv [12, 13], and the fully human scFv in C-CAR066 cells [37]. The Leu16 CAR-T cells were also among the first in the field to be evaluated in a human clinical trial [12]. Clinical results demonstrated safety, feasibility and potential efficacy of CAR-T cell therapy. Dr. Chen’s lab has developed a CD20 CAR-T cell construct with a rituximab-derived scFv and CD28 co-stimulatory signaling domain [21]. This construct had limited antitumor efficacy in comparison to FMC63-derived CD19 CAR-T cells [21]. By creating a hybrid scFv of the rituximab and Leu16 clone or inserting a torsional linker in the rituximab-derived CAR they altered the CAR confirmation which improved efficacy [21]. In contrast, we used the rituximab-derived scFv together with the 4-1BB signaling domain in our human CAR20-T construct. In our study, CAR20-T cells displayed efficient and specific cytotoxic potential against multiple CD20-expressing lymphoma cells in vitro. In the clinic, CD20-targeting antibody therapy rarely alters CD20 expressions, not even when resistance develops [10, 20]. In line with this, our CAR20-T cells displayed cytotoxicity against primary mantle cell lymphoma cells from a patient who relapsed after R-CHOP despite targeting the rituximab epitope. Even though the tumor cell killing is performed ex vivo, this result indicated clinical feasibility for our CAR20-T cells.

Selection pressure from CAR20-T cell therapy would most likely lead to modification or loss of CD20 as commonly seen for CD19 after CD19 CAR-T cell therapy [7, 10]. To overcome antigen loss, CAR-T cells simultaneously targeting multiple antigens have been developed [38–40]. However, dual antigen loss has also been observed [40, 41] indicating the need for new approaches to prevent relapses due to antigen loss. We have previously shown that arming CAR-T cells with inducible secretion of the bacterial-derived immune-stimulating factor NAP can induce bystander immunity and epitope spread, thereby eliminating CAR-target-negative tumor cells [27]. In this study, murine mCAR20(NAP)-T cells could slow down tumor growth and prolong survival in lymphoma-bearing mice without inducing toxicity. Improvement of mCAR20-T cell therapy upon incorporation of NAP implies that the therapy could be evaluated for patients with CD20-positive refractory or relapsed (r/r) B cell lymphomas, possibly also after relapse from CD19 CAR-T cell treatment.

No visible symptoms or weight loss were observed after mCAR20- or mCAR20(NAP)-T cell therapy indicating a safe profile of mCAR20(NAP)-T cell treatment. However, since the targeting moiety of the rituximab-derived CAR is against human CD20, the potential toxicity associated with systemic NAP release, following interaction with healthy B cells, could not be assessed in this murine model. Therefore, the safety of NAP armed CAR-T cells was measured using murine CAR19-T cells that target murine CD19 in immunocompetent mice with wild-type A20 lymphoma. This model aimed to capture any potential systemic toxicity from NAP. Histopathological examination did not show any lesions in major organs, except for the heart, which displayed mild lesions suggesting potential myxomatous degeneration of the cardiac valves. It has been reported in some studies that CAR-T cell therapy can affect the cardiovascular system [7, 42]. Notably, mild cardiac valves degeneration was observed in all groups, including the Mock-T cell treated group, indicating that it was not CAR-T cell or NAP related. Notably, the tissue sections were analyzed exclusively with HE-staining by an experienced pathologist. Additional staining of cell markers like CD3 can be performed to confirm these results. Moreover, given the absence of significant differences in liver enzymes and cytokines between the treatment groups, it is unlikely that CAR(NAP)-T will cause increased liver toxicity. Taken together, our results indicate that the safety profile of NAP-armed CAR-T cells is acceptable.

We further investigated the possibility of NAP-induced systemic toxicity in human blood using an ex vivo blood loop assay. This assay includes a specifically designed cytokine panel that can be applied in a clinical setting to predict adverse events like cytokine release syndrome (CRS) [43]. NAP did not induce lymphocyte activation as compared to Alemtuzumab. NAP increased the plasma levels of IL-6, IL-8 and TNFα, which have all been implicated in CRS [44], in a concentration-dependent manner. Alemtuzumab, which is approved for the treatment of chronic lymphocytic leukemia and multiple sclerosis, frequently induces CRS which can be managed by corticosteroid treatment [32, 45]. As NAP induces lower levels of cytokine release compared to Alemtuzumab, we predict that systemic cytokine release induced by NAP should be manageable in the clinic.

In summary, we have developed efficient CD20-targeting CAR-T cells that once armed with NAP display improved therapeutic potential in vivo without altering the safety profile. NAP further displayed a safe profile in a human blood loop assay. This study paves the way for further evaluation of the CAR20(NAP)-T in patients with refractory and relapsed B cell lymphoma.

## Materials and methods

### Cell lines and primary cells culturing conditions

The human lymphoma cell lines DB, and DG-75 [46] (Kind gift from Dr G Klein, Karolinska Institute, Stockholm, Sweden), U-698 [47] (Kind gift Kenneth Nilsson, Uppsala University), Daudi (ATCC CCL-213), Raji (Sigma Aldrich) were cultured in RPMI 1640 supplemented with 10% heat-inactivated fetal bovine serum (FBS), 1% penicillin/streptomycin (PeSt) and 1% sodium pyruvate. All the reagents were from Invitrogen. All lymphoma cell lines were modified to stably express green fluorescent protein, firefly luciferase (FLuc) and puromycin (for selection) by lentiviral transduction (Magnus Essand, Addgene, Plasmid #80,389) [48]. Cells expressing transgenes were selected using 5 µg/ml puromycin.

The mouse B cell lymphoma cell line A20 (ATCC TIB-208) was cultured in culture medium (RPMI 1640) containing 10% heat-inactivated FBS, 1% (PeSt) and 1% sodium pyruvate. To generate A20 cells stably expressing human CD20 (termed as A20-hCD20), A20 cells were transduced with a lentiviral vector encoding hCD20, fire-fly luciferase (FLuc) and puromycin separated by a self-cleaving peptide (T2A and F2A). A20-hCD20 cells were selected using 3 µg/ml puromycin.

Peripheral blood mononuclear cells (PBMCs) were isolated by Ficoll-Paque (GE Healthcare Life Science) from fresh buffy coats taken from healthy donors, collected from the Blood Center at Uppsala University Hospital. They were used as a source for human T cells, which were cultured in RPMI 1640 supplemented with 10% FBS, 1% PeSt and 1% sodium pyruvate with 50 IU/ml IL-2 (Proleukin, Novartis) if not stated otherwise.

### Retro- and lentiviral vector construction and production

To create a lentiviral vector encoding CAR20 the variable region sequence from rituximab (PDB: 4KAQ) was incorporated into a CAR cassette with the transmembrane region from CD8 and the signaling domain from CD3zeta and the co-stimulatory signaling domain from CD137 as previously described [49]. The CAR constructs were cloned into a third-generation self-inactivating (SIN) lentiviral vector (SBI, System Biosciences) under the control of elongation factor-1 alpha (EF1a) promoter. Green fluorescence protein (GFP) was inserted after the CAR construct using a T2A self-cleaving peptide in both plasmids to allow detection of CAR-T cells. Thy1.1 is used as a surrogate marker for detection after the CAR construct in some cases. A mock construct only encoding GFP under the control of the EF1a promoter was used as a control. All sequences were purchased from GenScript. Production of third generation viral particles has been previously described [50]. The retroviral vector for engineering human CAR20(NAP)-T was purchased from BioNTech IMFS (Idar-Oberstein, Germany) and used in the in vivo efficacy studies (xenograft Daudi model).

To generate murine CAR constructs, the variable region of rituximab was incorporated into a murine CAR backbone containing murine CD3ζ.1-3 and murine CD28 transmembrane and signaling domains, as described previously [51]. To construct retroviral vector RV(mCAR20), the above constructs were subcloned into mouse stem cell virus-based retroviral vector, pMIG-W (Luk Parijs, Addgene, plasmid #12,282) [52]. The RV(mCAR19) and RV(mCAR19-NAP) constructs have been described previously [27]. An empty retroviral vector was used as Mock control. The NAP sequence was inserted in the orientation opposite the CAR20 cassette and under the control of murine NFAT-IL-2 promoter to construct retroviral vector RV(mCAR20-NAP). Retrovirus was produced using the packaging plasmid pCL-Eco and the Gryphon-Eco retroviral packaging cell line (Allele Biotechnology).

### Human T cell engineering, enrichment and expansion

Human PBMCs were activated using OKT-3 (100 ng/ml, BioLegend San Diego, CA) or TransAct (1:100, Miltenyi Biotec) and IL-2 (100 IU/ml, Proleukin) for 3 days at a concentration of 2 × 10^6^ cells per ml. Activated T cells (1 × 10^6^ cells) were re-suspended in 20 µl concentrated lentivirus together with 10 mg/ml protamine sulfate (Sigma-Aldrich) and IL-2 (100 IU) and incubated for 4 h at 37 °C. The following day, T cells were transduced in the same manner. After transduction, T cells were cultured (in culture medium supplemented with 50 IU/ml IL-2) for 7 days before CAR-T cells were enriched by sorting out GFP^+^ cells (BD FACSAriaIII, BD Biosciences). Sorted cells were expanded using a rapid expansion protocol previously described [53].

### Mouse T cell activation and transduction

Spleens were collected from Balb/c mice (Janvier) and mashed against a 70 µm cell strainer (Corning) to create single cell suspension. After resuspended in red blood cell lysis buffer (ACK lysing buffer, Invitrogen) for 5 min, splenocytes were washed and activated using Concanavalin A (2 µg/ml) (Sigma Aldrich) and murine IL-7 (1 ng/mL) (Pepro Tech) in RPMI 1640 containing 10% FBS, 1% PeSt and 1% sodium pyruvate. Two days post activation splenocytes were transduced with retrovirus as previously described [27].

### T cell cytotoxicity assay and ELISA

#### Human T cell cytotoxicity assay

The cytotoxicity assay was performed using luciferase-expressing target cells. CAR-engineered human T cells were rested for 3 days after rapid expansion in a medium containing a low dose of IL-2 (20 IU/ml) before performing the cytotoxicity assay. The T cells were co-cultured with luciferase-tagged target cells (DG75, DB, U698, Raji and Daudi, at 3:1 ratio) in a total volume of 200 μl, in a round-bottom 96-well plate. To detect the luciferase expression and activity, ONE-Glo luciferase assay system (Promega) was added according to manufacturer’s protocol. Specific lysis of target cells was calculated by comparing luminescence of co-cultured samples to untreated target cells.

#### Murine T cell cytotoxicity assay

The cytotoxicity assay was performed using luciferase-expressing target cells. Murine splenocytes were harvested, activated, and transduced with retroviral vectors as described above. The engineered-T cells were co-cultured with firefly luciferase-tagged A20 or A20-hCD20 target cells at different ratios (20:1, 5:1 and 1:1) in a round bottom 96-well plate (total volume 200 µl). After 5 days of co-culture, 80 µl supernatant was collected for IFN-γ and TNF-α analysis using an ELISA kit (Mabtech). Luciferase expression and activity (as an indicator of target cell viability) were determined by using ONE-Glo luciferase assay system (Promega) according to manufacturer’s protocol. Specific lysis of target cells was calculated by relating luminescence of co-culture with target cells alone.

#### T cell cytotoxicity assay towards patient samples

Biopsy samples containing primary mantle cell lymphoma (MCL) cells were either left untreated or co-cultured together with engineered-T cells at a 1:1 (1 = 25 000 cells) ratio for 3 days in a round bottom 96-well plate in a total volume of 200 µl per well. After 2 days of co-culture, supernatant was collected for analysis of IFN-γ secretion by an ELISA kit (Mabtech). After 3 days of co-culture, the remaining amount of CD20^+^ cells (as an indicator of target cell viability) was assessed by flow cytometry. Specific lysis of target cells was shown as the proportion of CD20^+^ cells normalized to the one at the start of the experiment.

#### Cytotoxicity assay using incucyte

Measurement of immune cell killing of target tumor cells was analyzed with the Incucyte® Zoom image analysis software (Essen BioScience). Briefly, one image per well in a 24-h repeat scanning was scheduled with the Phase contrast, green and red channels in 10 × objective. Images were taken every hour with the standard scan schedule and Basic Analyzer module. Image collections and processing definitions for every cell line and assay were established following the suggested procedures by the manufacturer. Processing definitions were established with phase and fluorescence masks for scarlet expression to quantify target cell proliferation, determined as total red fluorescence confluence. Target cell killing by CAR-T cells was determined by comparing red fluorescent signal of co-cultured (at the effector to target ratio 5:1) samples to untreated target cells and reported as relative cell viability.

### Flow cytometry

To assess transduction efficiency, CAR and GFP expression of engineered T cells were detected by flow cytometry. The T cells were stained with CD3-BB700 (Clone SP34-2) and anti-mouse IgG (H + L)-AF647 (to detect CAR expression, A21237, Thermo Fisher Scientific).

Human CD20 expression in human cell lines and in murine cell lines A20 and modified A20-hCD20 were assessed by staining the cells with anti-human CD20-APC (Clone 2H7). An isotype was used as control (Mouse IgG2b, κ). Stained cells were analyzed by flow cytometry using BD FACSCanto II (BD Biosciences) or CytoFLEX LX (Beckman Coulter) and analyzed using FlowJo_v10.8.1 (FlowJo LLC).

### Animal experiment

#### Survival experiment

Daudi model: NOD-SCID mice (8 weeks) were implanted intravenously (i.v.) with 1 × 10^6^ firefly luciferase (fLuc) expressing Daudi cells on day 0. Mice were then treated with two doses (2 × 10^6^ cells/dose) intravenously on days 4 and 8 post tumor implantation. The tumor progression was monitored by in vivo imaging system (IVIS) Lumina III (PerkinElmer). Mice were intraperitoneal (i.p.) injected with 100 μL of 30 mg/mL of D-Luciferin potassium salt (Perkin Elmer) and imaged 10 min after substrate injection. Mice were monitored regularly for their health status and euthanized when reaching the humane endpoint.

A20-hCD20 model: Female Balb/c mice (8 weeks) were implanted subcutaneously (s.c.) with 2 × 10^5^ murine lymphoma A20 expressing human CD20 (A20-hCD20) on day 0. The mice were treated with 3 × 10^6^ T cells intravenously (i.v.) 10-, 14-, and 18-days post tumor implantation. The mice were monitored regularly and euthanized when reaching the humane endpoint. Mice were sacrificed when reaching the humane endpoint (tumor size of 1cm^3^). The experiment was performed twice, and all data were pooled together (Mock-T: n = 20, mCAR20-T: n = 20, mCAR20(NAP)-T: n = 19).

The mice were monitored for tumor growth until reaching the humane endpoint volume 1000 mm^3^. The tumor size was calculated as: volume = length × width^2^ × π/6. The time to endpoint (TTE) for each mouse was calculated as TTE = [log (EPV)-b]/m. The constant b is the intercept and m are the slope of the line obtained by linear regression (time vs tumor volume) of a log-transformed tumor volume data set, which comprised of the first measured tumor volume when EPV was exceeded and three consecutive tumor volumes immediately prior to the attainment of EPV. Any animal that died from treatment-related causes was assigned a TTE value equal to the day of death. Any animal that died from non-treatment-related causes was excluded from the analysis. Survival curve was generated based on the TTE value using the Kaplan–Meier method and compared using the log-rank (Mantel-Cox) test.

#### Safety assessment

In experiment setting #1: Female Balb/c mice (3 mice/group) were subcutaneously implanted on day 0 with A20 tumor (murine lymphoma cell line A20) and treated intravenously with Mock-T, mCAR-T or mCAR(NAP)-T cells (targeting murine CD19) on days 10, 14 and 18. Different organs (brain, heart, kidney, lung, liver, and spleen) were collected on day 20, and fixed in 4% formalin, embedded paraffin, and sent to BioVet AB (Sollentuna, Sweden) for pathologic evaluation. Sections were made from these samples with 3–4 µm thickness and stained with Hematoxylin–Eosin (HE). Representative images were taken from all examined organs.

In experiment setting #2: Female Balb/c mice (3 mice/group) were subcutaneously implanted on day 0 with tumor cells (murine lymphoma cell line A20) and treated intravenously with Mock-T, mCAR-T, or mCAR(NAP)-T cells (targeting murine CD19) on day 10. Blood was collected on day 3, 12, 18, and 28 for measurement of systemic cytokines and liver enzymes. A panel of 13 cytokines in blood samples were measured using LEGENDplex (BioLegend, Cat.: #741,023), and read-out on Cytoflex FL following the manual. Liver enzyme levels were measured at Clinical Chemistry Department in the Animal Hospital at the Swedish University of Agricultural Science (Uppsala, Sweden).

### Whole blood loop analyses (Immuneed AB)

Recombinant NAP at two different concentrations were evaluated based on the following assumptions: 1) a patient will receive up to 2 × 10^8^ CAR^+ ^T cells; 2) all CAR-T cells expand 1000-fold, and all cells release NAP; 3) NAP is expressed at 1 × 10^6^ molecules per cell. This is estimated based on T cell volume of 200 femtoliter [54], and a density of 2.7 × 10^6^ total protein molecules per 1 femtoliter cell volume [55] wherein 1/500 is NAP. These estimations resulted in a total of 2 × 10^17^ NAP molecules. With an estimated average blood volume of 5000 mL, the maximum NAP concentration in circulation would be 2.3 µg/mL. We therefore tested 2 × and 10 × that dose level in the blood loop assay.

Fresh whole blood was taken from ten healthy volunteers (D1-D10) and mixed immediately with a low amount (2 IU/mL) of soluble heparin. Directly after, whole blood was transferred into a plastic tubing loop, which was pre-coated with heparin. followed by administration of the test items, 5 μg/ml NAP proteins and 25 μg/ml NAP proteins. PBS was used as negative control, 3 μg/ml Alemtuzumab (Lemtrada, Sanofi) was used as positive antibody, and 3 μg/ml LPS (Sigma-Aldrich, Sweden) was used as positive control. Additionally, fresh blood from each donor was used for hematology measurements directly after blood collection (described as pre-loop baseline). Additionally, blood collected directly from donors was processed into plasma and included cytokine and complement analysis. Blood samples were collected at 15 min for complement analysis and immune cells activation, while blood samples collected at 4 h were analyzed for hematology analysis, immune cell activation, and cytokine analysis.

For hematology analysis, parameters included: red blood cell (10^12^/L), platelets (10^9^/L), white blood cell (10^9^/L), differential neutrophil (10^9^/L), lymphocyte (10^9^/L), monocyte (10^9^/L), eosinophil (10^9^/L) and basophil (10^9^/L). These parameters were measured at the time points 0 h (pre-loop baseline) and 4 h by the Hematology Analyzer Sysmex XN-L350 (Sysmex). Hematology analysis of time point 0 h was performed as three subsequent measurements, while samples collected in the loop system at 4 h were done as single measurements.

For cytokine and complement analysis, plasma harvested at the time point 0 h (pre-loop baseline) and 4 h were analyzed for IFN-γ, TNF-α, IL-2, IL-6 and IL-8 with a Mesoscale V-plex kit (Meso Scale Discovery), according to the manufacturer’s instructions. Complement analysis (C3a and C5a) was performed on plasma collected at 0 h (pre-loop baseline) and 15 min time-point with ELISA kits (Raybiotech), according to the manufacturer’s instructions. Limit of detection (LOD) is the smallest concentration of an analyte from which it is possible to deduce the presence of an analyte in the test sample, while limit of quantification (LOQ) is the smallest concentration of an analyte that can be determined with the specified degree of accuracy and precision. Lower LOD (LLOD) was calculated by MSD software and defined as 2.5xSD above the zero calibrator (Standard-8).

For immune cell activation, blood was collected at the time points 0 min, 15 min, and/or 4 h time points, and the activation of immune cells were analyzed by flow cytometry (CytoFLEX LX, Beckman Coulter). The platelets were defined as CD41^+^ CD45^−^ cells and the activation marker CD62P was analyzed. The lymphocytes were first identified in the forward versus side scatter (FSC/SSC) plot and further gated as T cells (CD3^+^CD56^−^), NK cells (CD3^−^CD56^+^), and B cells (CD3^−^CD19^+^). The activation phenotype of lymphocytes was determined by staining CD69 and CD107a. Granulocytes and monocytes were also identified in the FSC/SSC plot and further defined as granulocytes (CD14^−^CD66b^+^) and monocytes (CD14 + CD66b^−^), respectively. The activation markers were analyzed by staining CD11b and CD83.

### Ethical approval

The Uppsala Research Animal Ethics Committee has approved all the animal studies (5.8.18–19,434-2019). The human buffy coats obtained from healthy donors were anonymized. The patient-derived mantle cell lymphoma cells were collected under ethical permit: EPN 233–2014.

### Statistical analysis

All statistics were performed in GraphPad Prism. For statistical analysis between two experimental groups, a non-parametric unpaired t-test was used to compare means between experimental groups. Log-rank test was used to compare the Kaplan–Meier survival curve. For experiments containing more than 2 groups, either One-way ANOVA or Two-way ANOVA were used, and the exact methods are indicated in each figure legend. P-value of less than 0.05 was considered as statistically significant.

## Supplementary Information

Below is the link to the electronic supplementary material.

Supplementary file1 (PDF 10860 kb)

## Data Availability

No datasets were generated or analysed during the current study.
