# Supplementary material for: Pre-clinical safety and efficacy evaluation of Helicobacter Pylori neutrophil-activating protein (NAP)-armed CAR-T cells targeting B cell lymphomas
Source: Cancer Immunol Immunother. 2025 Jul 12;74(8):262. doi: 10.1007/s00262-025-04112-1 (PMC12255594; doi:10.1007/s00262-025-04112-1)
Supplement: Supplementary file 1 — Supplementary file1 (PDF 10860 kb) [file 262_2025_4112_MOESM1_ESM.pdf]

Supplementary Figure S1

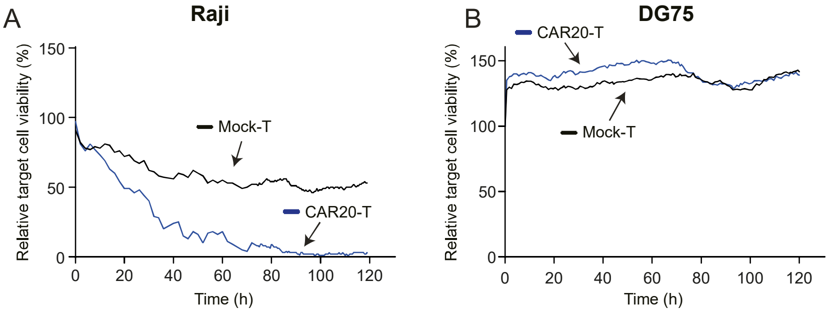

Supplementary Figure S1. Incucyte assay to evaluate CAR20-T cell killing potency of lymphoma cell lines. (A-B) Relative viability of scarlet-expressing (**A**) Raji or (**B**) DG75 cells measured every hour over time by total integrated intensity using Incucyte after co-culture with CAR20-T cells or Mock-T cells, prepared from one healthy donor, at the effector to target ratio 5:1.

Supplementary Figure S2

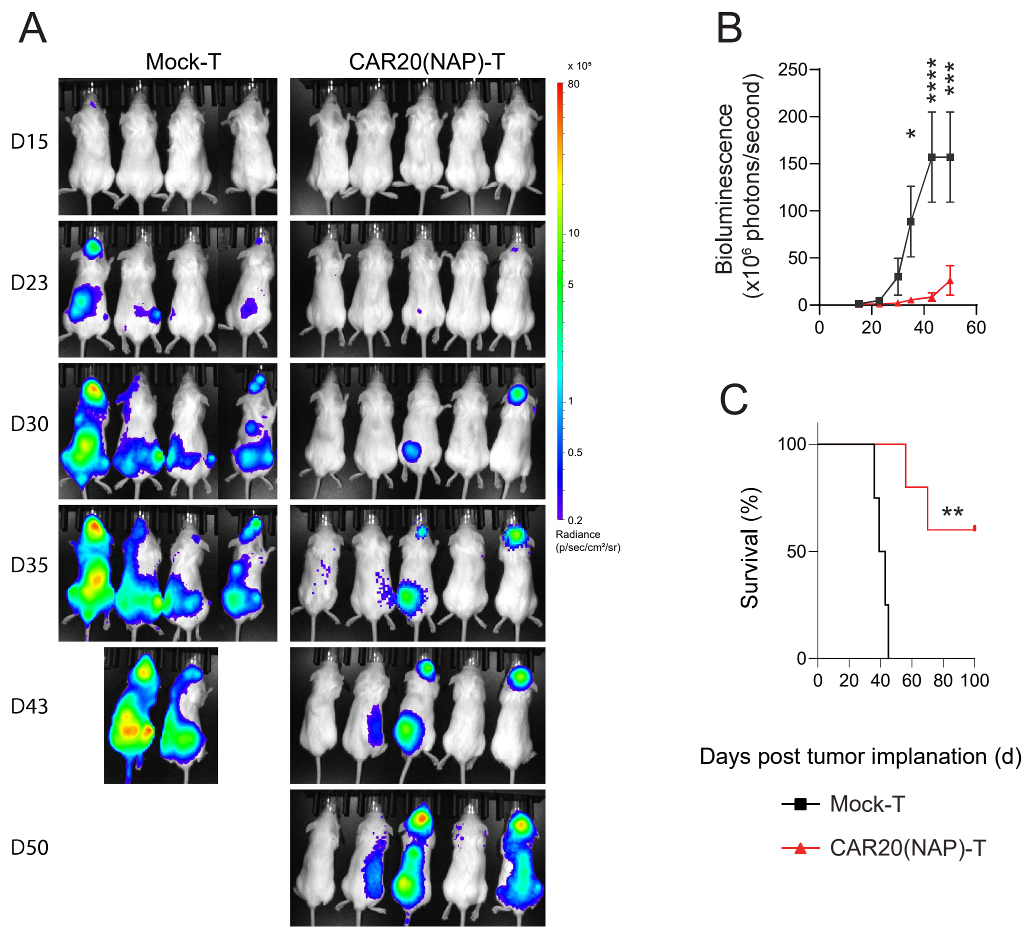

Supplementary Figure S2. CAR20(NAP)-T cells prolong survival of NOD-SCID mice with systemically injected human lymphoma cells. NOD-SCID mice were injected intravenously with 1×10^{6} firefly luciferase (fLuc) expressing CD20^{+} Daudi cells on day 0. Mice were then treated with two injections (2×10^{6} cells/dose) of Mock-T cells (n=6) or CAR20(NAP)-T cells (n=5) on days 4 and 8. Tumor progression was monitored by bioluminescence imaging. (**A**) Representative bioluminescence images of individual mice after treatment at different time points as indicated. (**B**) Tumor burden presented by bioluminescence (in photons/second). Two-way ANOVA was performed with Sidak correction for multiple comparisons bwtween groups at each time point (*p<0.05; ***p<0.001; ****p<0.0001). (**C**) Kaplan-Meier survival curve. The survival curves were compared by Log-rank test (**p<0.01).

Supplementary Figure S3

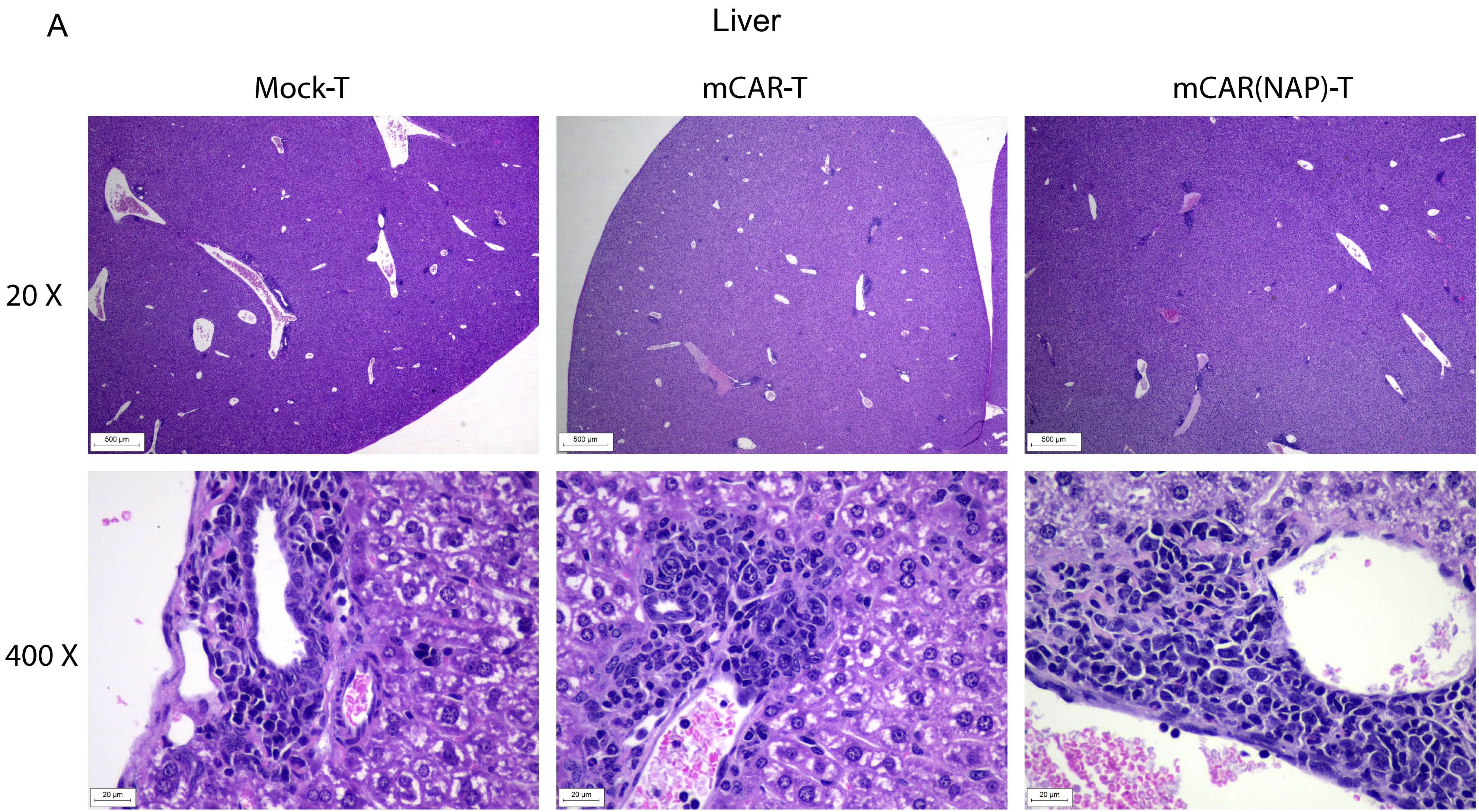

Supplementary Figure S3. Histopathology analysis of major organs indicate no tissue damage. Representative images of hematoxylin-eosin (HE) stained tissue sections from (**A**) Liver, (**B**) Heart, (**C**) Brain, (**D**) Lung, and (**E**) Spleen. Images were taken at different magnifications and locations of tissue as indicated.

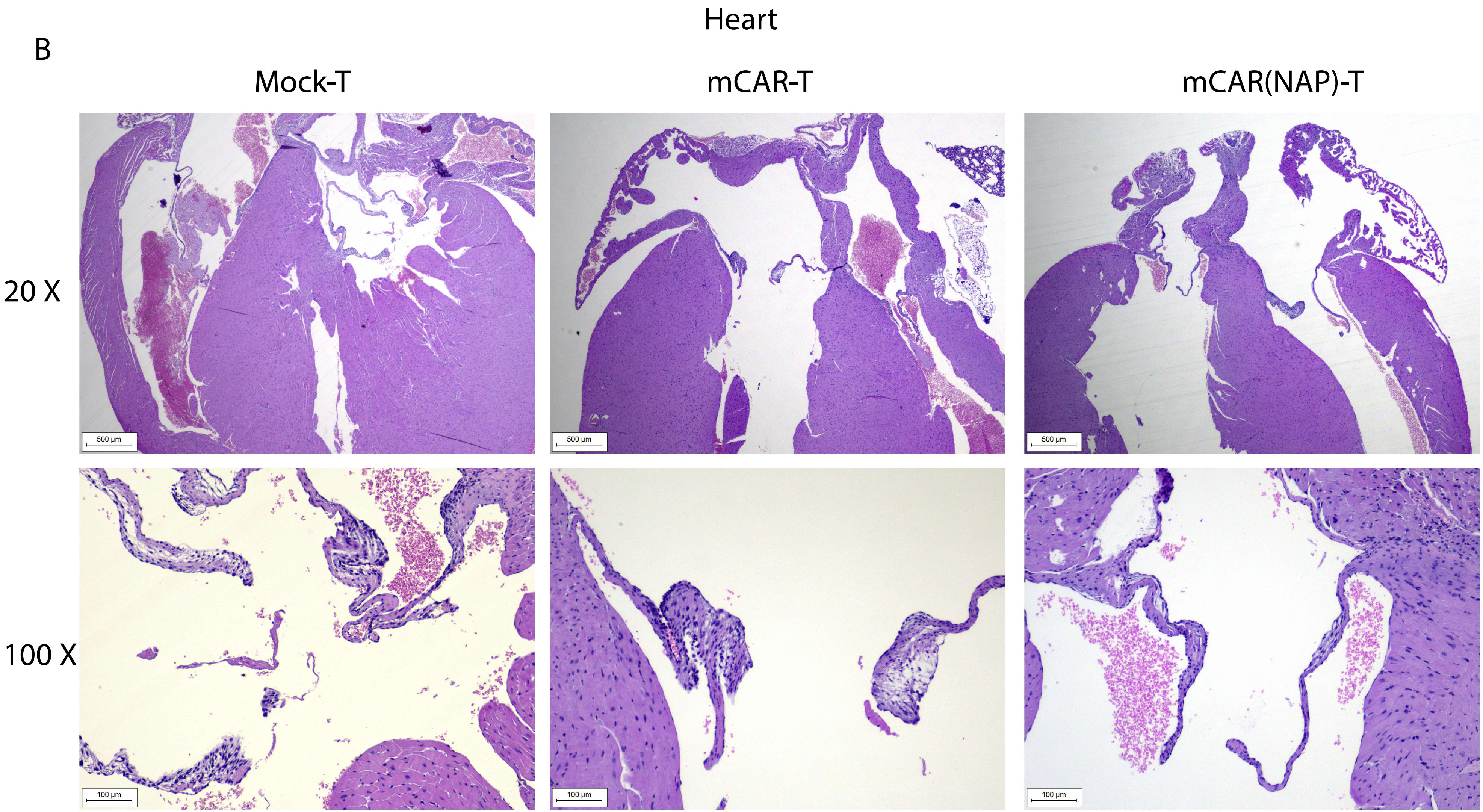

B
Heart
Mock-T
mCAR-T
mCAR(NAP)-T
20 X
100 X
500 µm
500 µm
500 µm
100 µm
100 µm
100 µm

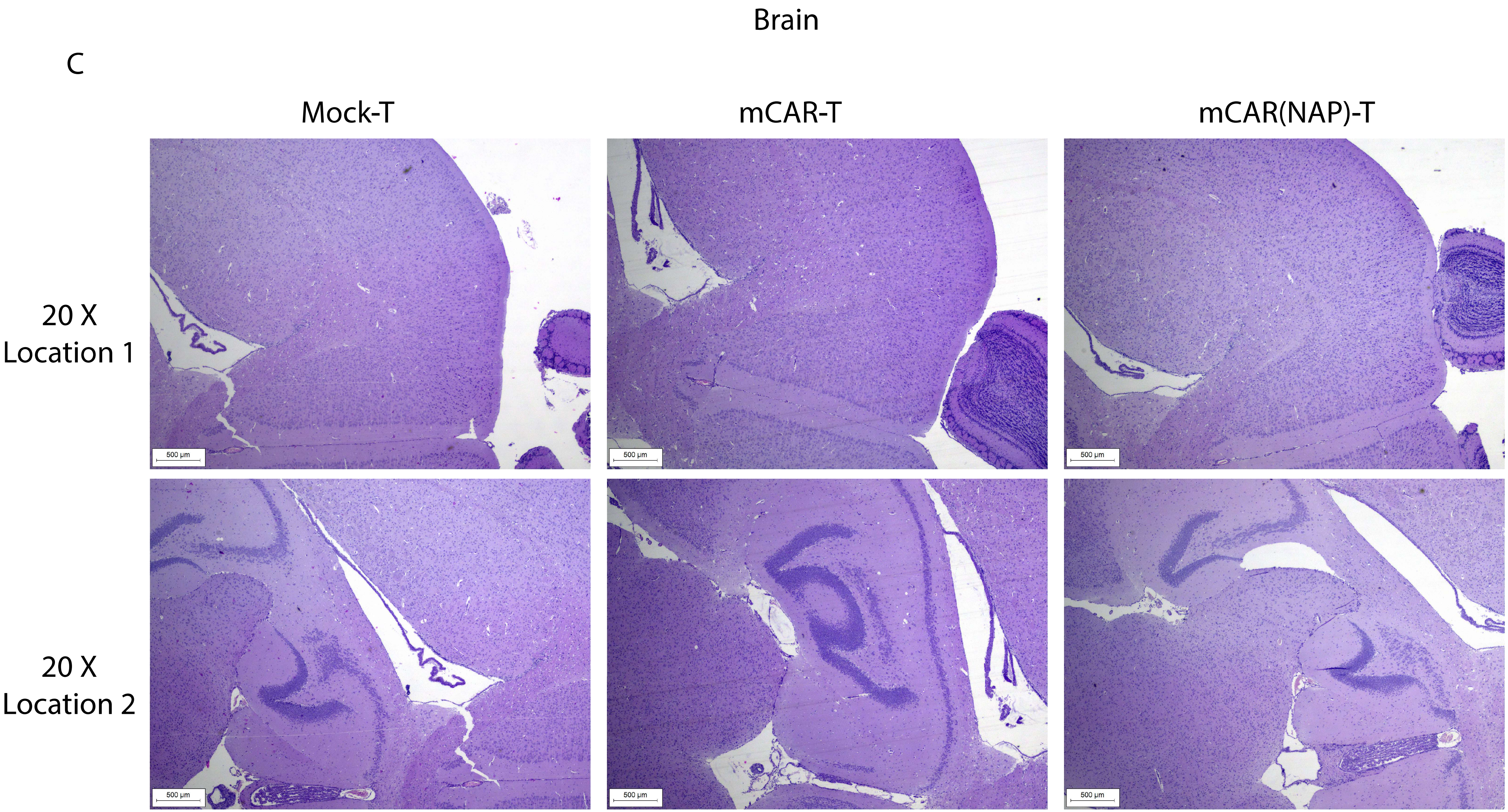
Brain
C
Mock-T
mCAR-T
mCAR(NAP)-T
20 X
Location 1
20 X
Location 2
500 µm

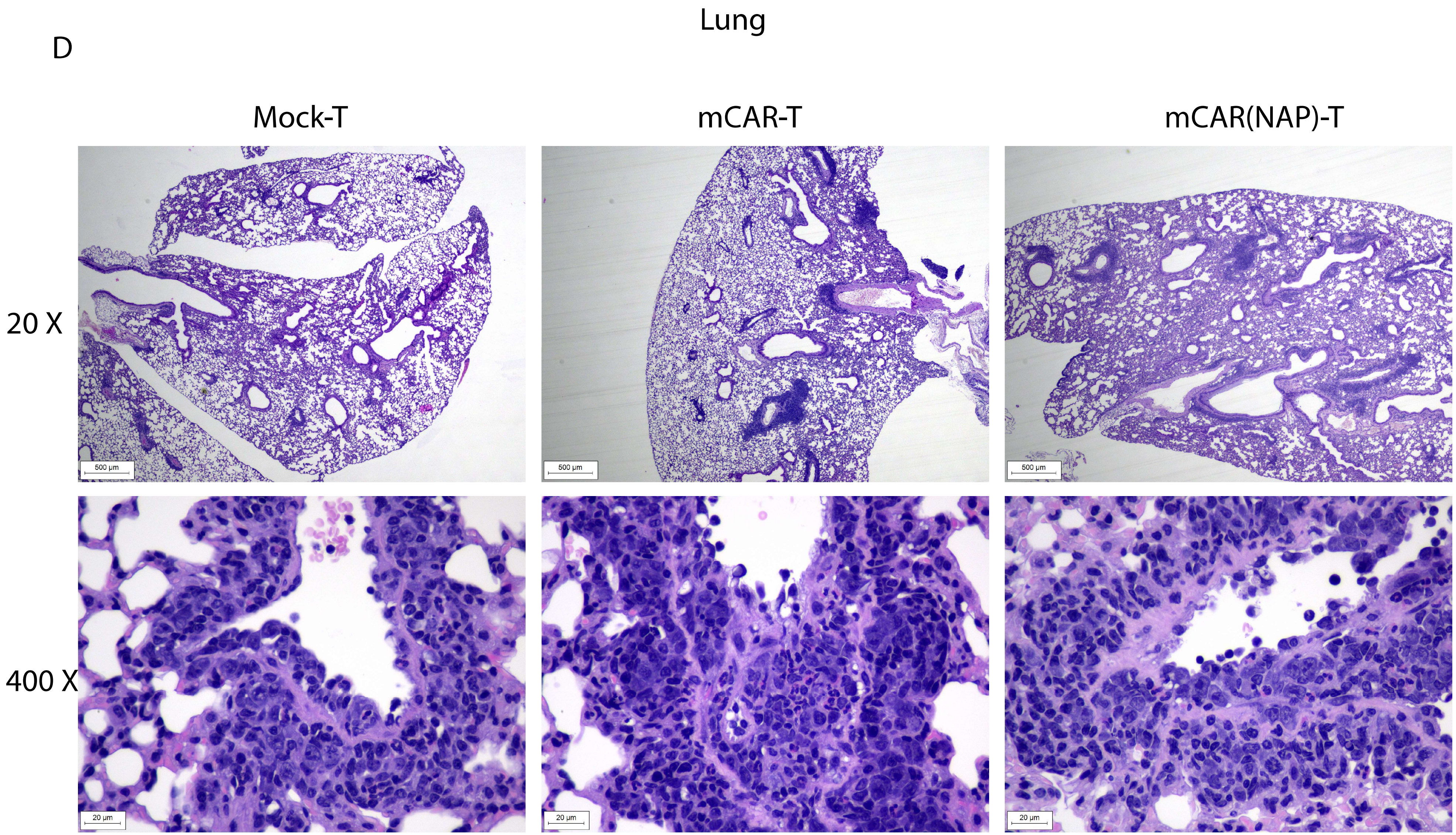
D
Lung
Mock-T
mCAR-T
mCAR(NAP)-T
20 X
400 X
500 μm
20 μm

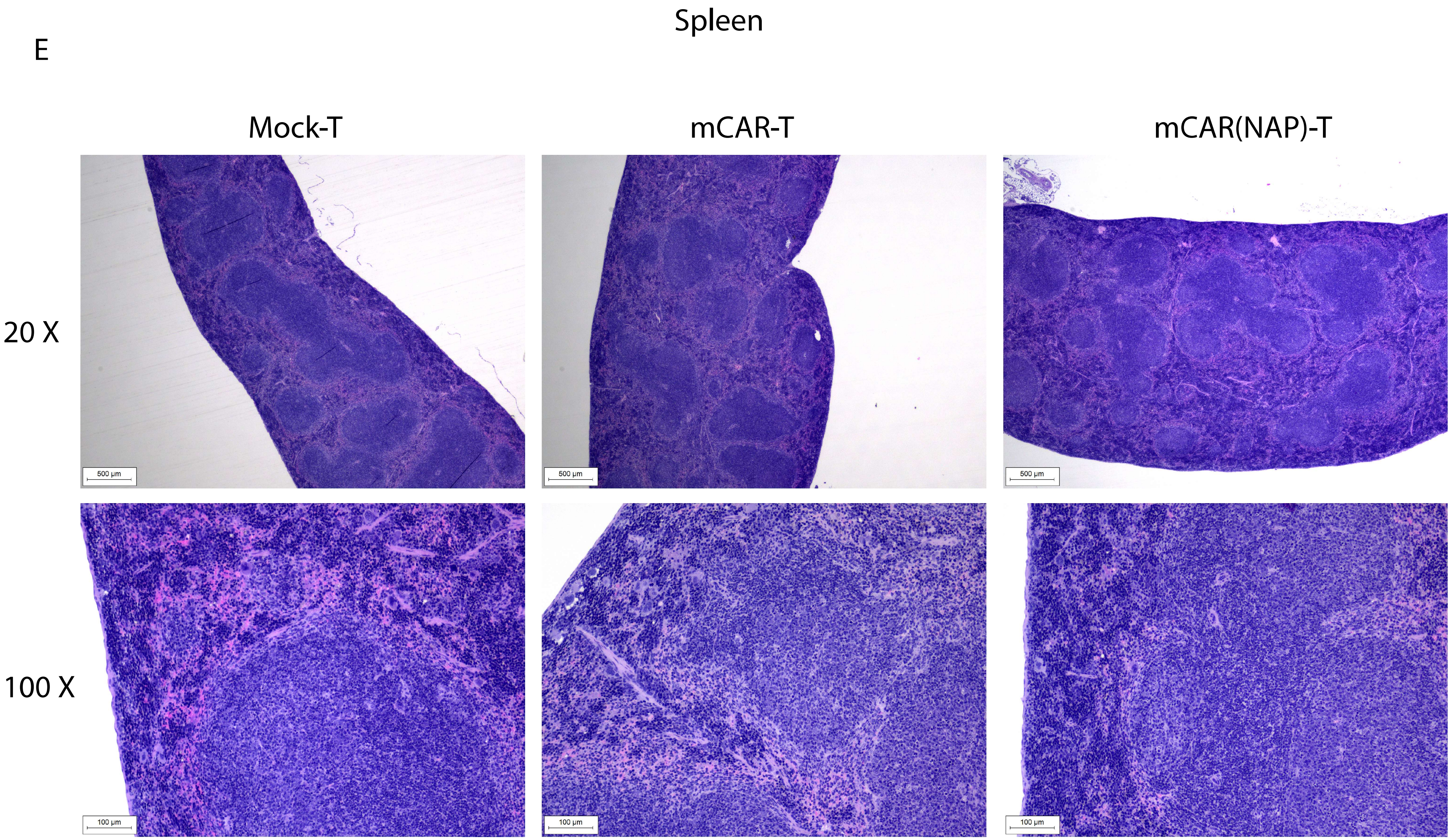
E
Spleen
Mock-T
mCAR-T
mCAR(NAP)-T
20 X
100 X
500 μm
100 μm

Supplementary Figure S4

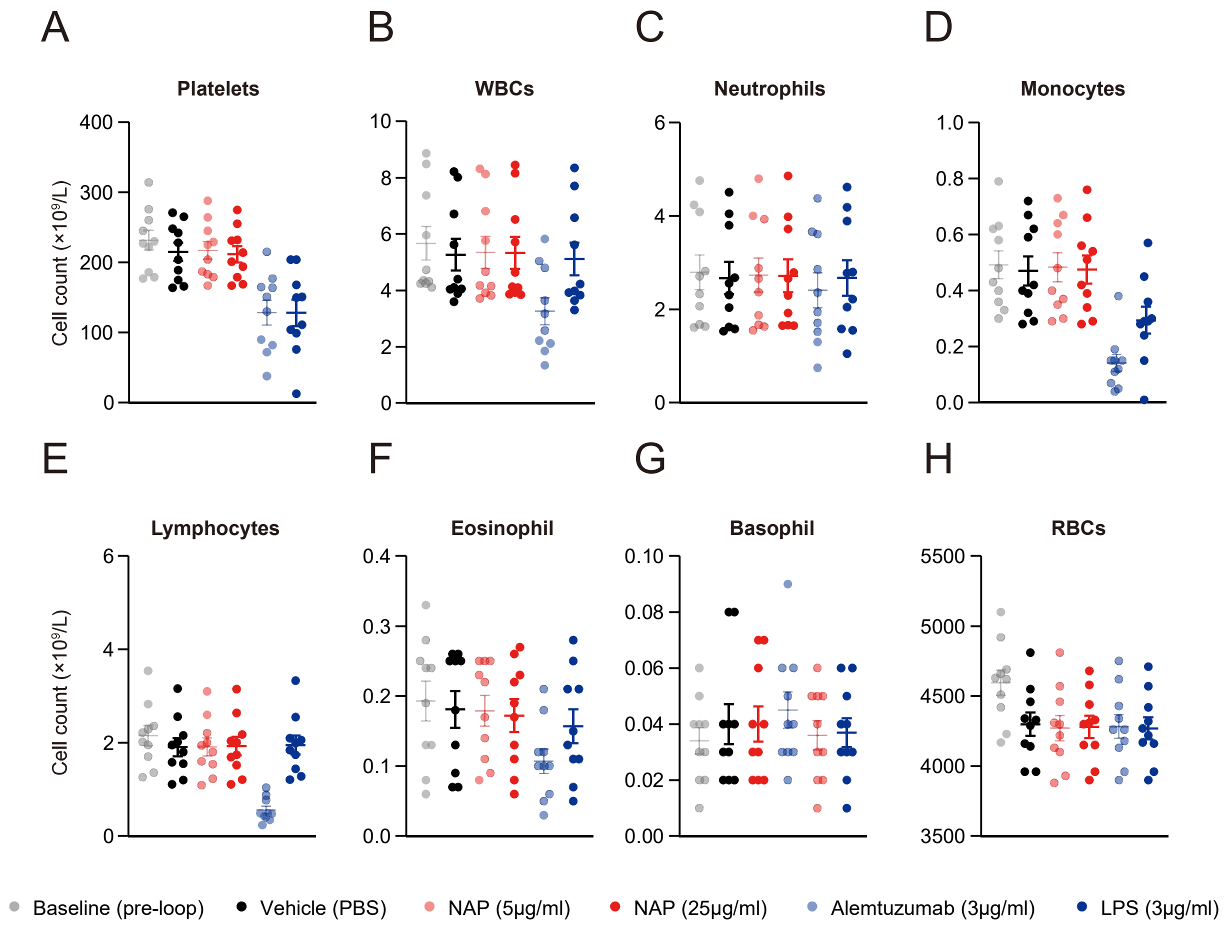

Supplementary Figure S4. Hematology analysis of human blood components after exposure to recombinant NAP protein in a human whole blood loop system. Freshly acquired whole blood was incubated with PBS, NAP proteins, Alemtuzumab or LPS in a circulation loop system. The final concentrations of the NAP proteins, Alemtuzumab and LPS were displayed in brackets in the figure. After 4h, blood samples were collected analyzed for hematology parameters, including (**A**) platelets, (**B**) white blood cell count WBCs, (**C**) neutrophils, (**D**) monocytes, (**E**) lymphocytes, (**F**) eosinophil, (**G**) basophil and (**H**) red blood cell count RBCs.
